# High Prenatal Exposure to Bisphenol A Reduces Anogenital Distance in Healthy Male Newborns

**DOI:** 10.4274/jcrpe.4817

**Published:** 2018-02-26

**Authors:** Emil Mammadov, Murat Uncu, Ceyhun Dalkan

**Affiliations:** 1 Near East University Faculty of Medicine, Department of Pediatric Surgery, Nicosia, Cyprus; 2 Near East University Faculty of Medicine, Department of Biochemistry, Nicosia, Cyprus; 3 Near East University Faculty of Medicine, Department of Pediatrics, Nicosia, Cyprus

**Keywords:** Anogenital distance, bisphenol A, cord blood, newborn

## Abstract

**Objective:**

To estimate the relationship between cord blood bisphenol A (BPA) levels and anogenital measurements in healthy newborns.

**Methods:**

Pregnancy and birth history, together with body mass and length data, anogenital measurements, penile measurements and cord blood samples were obtained from healthy newborns. Cord blood concentration of BPA was analyzed by sandwich enzyme-linked immunosorbent assays kit.

**Results:**

Among 130 healthy newborns (72 boys, 58 girls), mean anopenile distance was 45.2±6 mm and anoscrotal distance was 21.9±5.4 mm in boys; mean anoclitoral distance was 33.8±6.6 mm and mean anofourchette distance was 12.2±4.9 mm in girls. Mean cord blood BPA level was 4.75±2.18 ng/mL. 90^th^ percentile value for cord blood BPA was 8.26 ng/mL and the analysis showed a statistically significant correlation between anoscrotal distance and cord blood BPA levels above the 90th percentile (p=0.047) in boys. The changes in anogenital distance in girls were not statistically significant.

**Conclusion:**

We showed a significant association between high cord blood BPA levels and shortened anoscrotal distance in male newborns. However, this result should be interpreted with caution since there were no significant external genital abnormalities in our study group.

## What is already known on this topic?

Bisphenol A (BPA) is suspected to alter genital development. Several animal studies have shown significant results regarding this issue. At the same time, several human studies have reported significant results on the relationship between BPA and genital development.

## 

### What this study adds?

To our knowledge, this is the first study investigating the relationship between cord blood BPA levels and anogenital measurements in healthy newborns.

## Introduction

Being first produced as a synthetic estrogen, bisphenol A (BPA) was later widely used in the plastic and resin production industry as a plasticizer due to its cross-linking qualities. Exposure of products containing BPA to increased temperatures was shown to cause leakage of this molecule into food and beverages (1,2). BPA is detectable in many body fluids including blood, urine, amniotic fluid, breast milk and cord blood. The anti-androgenic effect of this chemical has been widely researched and is suspected to be related to several disorders, both in childhood and adulthood (3). The main emphases of current studies are the effect of BPA on obesity, gonadal abnormalities, infertility, thyroid function and malignancy (4,5,6,7,8,9,10,11). 

Anogenital distance (AGD) is a relatively new measurement parameter showing the distance from anus to the genitals. While it was used for sex determination in animals for a long time (12,13), the effort to implement this measurement as an epidemiological marker of genital development in humans is quite recent. Animal studies have shown a shortened AGD in male offspring reflecting decreased *in utero* androgen exposure and conversely a longer distance in females reflecting increased *in utero* androgen exposure (14). 

In this study, we aimed to investigate the relationship between cord blood BPA levels and anogenital measurements in healthy newborns.

## Methods

Near East Univestity Local Ethics Committee approval was obtained prior to the study (approval number: YDU/2015/32-215) and informed parental consent was obtained for each participant. One hundred and fifty healthy newborns up to three days of age who were born in the period of May-August 2016 were included in the study population. Infants who had congenital anomalies, perinatal asphyxia, major surgical operation and those who were hospitalized in the neonatal intensive care unit were excluded from the study. Pregnancy and birth history together with body mass and length data were obtained from the patients’ hospital records.

Cord blood samples from the umbilical vein were obtained at birth and collected into BPA-free polystyrene tubes (BD Diagnostics Preanalytical Systems, BE). Each blood sample was left to coagulate for 30 minutes, then samples were centrifuged at 2000 g for 10 minutes at room temperature to obtain serum, which was stored in aliquots in BPA-free Eppendorf (Eppendorf AG, GE) vials at -80 °C until analysis. On the day of analysis, the aliquots were brought to room temperature and thoroughly vortexed before the analysis. Total serum concentration of BPA was analyzed by sandwich enzyme-linked immunosorbent assays (ELISA) kit (General Bisphenol A ELISA, MyBioSource, Inc., San Diego, California, USA) with a Spectramax M5 Series Multi-Mode Microplate Reader (Molecular Devices, Sunnyvale, California, USA). The kit is characterized by a limit of detection for BPA of 0.6 ng/mL.

The anogenital measurement technique for the study was standardized as follows. The infants were placed in supine position, with flexed hips and knees to provide a “frog leg” posture. After marking the center of the anus with a pencil, the distances from the anus to the anterior base of the penis; anopenile distance (AGD_AP_) and to the base of the scrotum; anoscrotal distance (AGD_AS_) were measured in boys. In girls, the distance from anus to the anterior tip of the clitoral hood; anoclitoral distance (AGD_AC_) and the posterior fourchette of labia majora; anofourchette distance (AGD_AF_) were measured. Two blinded (digital screen turned away from the researcher) measurements per patient with digital Vernier caliper were obtained from five newborns by two different researchers as a pilot study to assure the right measurement technique. The study proceeded after the measurement consistency was ensured. All measurements were performed by one blinded researcher and the results were analyzed and interpreted by a second blinded researcher.

### Statistical Analysis

Statistical analysis was performed using SPSS version 22 for Macintosh (SPSS Inc., Chicago, Illinois, USA). The results are expressed as mean and standard deviation of the mean. To determine the relationship between principal variables and the other continuous variables, the Pearson correlation test was used. The Mann-Whitney U test was used to determine the relationship between grouped variables. A p value less than 0.05 was considered statistically significant.

## Results

Twenty newborns were excluded due to the exclusion criteria, leaving 130 patients in the study group. This consisted of 72 (55%) boys and 58 (45%) girls. The mean birth weight of the group was 3172±492 grams and mean birth length was 48.3±2 cm. In boys, the mean AGD_AP_ was 45.2±6 mm and AGD_AS_ was 21.9±5.4 mm. In girls, the mean AGD_AC_ was 33.8±6.6 mm and mean AGD_AF_ was 12.2±4.9 mm. Mean cord blood BPA level was 4.75±2.18 ng/mL (Table 1). The 90^th^ percentile value of the cord blood BPA was 8.26 ng/mL. None of the patients had any obvious genital development abnormality.

In general, anogenital measurements did not show statistically significant correlations with the cord blood BPA levels (p>0.05). However, a significant negative correlation was found between AGD_AS_ and cord blood BPA levels above the 90^th^ percentile (p=0.047) in boys. AGD_AS_ mean value was significantly lower in the group with cord blood BPA levels above 90^th^ percentile (higher than 8.26 ng/mL). In contrast, an apparent but not statistically significant increase was noted in AGD_AP_ with increased levels of BPA. In girls, a statistically nonsignificant increase in the AGD_AC_ and a similar decrease in the AGD_AF_ was found in the group with high cord blood BPA levels (Table 2).

## Discussion

BPA, a well-known endocrine disruptor has been investigated for more than a decade. Leaching of this molecule from daily used plastic products is highly dependent on heat, on contact with chemicals and deterioration of the product itself (15,16). Drinking water carries risk of pollution by BPA as plastic bottles used for household water dispensers are being reused and exposed to high temperatures during the cleaning process. BPA is suspected to have an anti-androgenic effect on genital development *in utero*. Studies regarding the prenatal effect of BPA on fetal development have mostly been performed in rodents and confirm adverse effects of increased BPA exposure on the growth and genital development of the offspring (14,17). The mechanism of action of BPA is thought to be through its binding to estrogen receptors thus triggering their activation. However, some authors do not attribute the anti-androgenic action solely to estrogen receptor activation as BPA has the ability to interact with other receptors such as aryl hydrocarbon receptor (the androgen receptor behaving as an antagonist) and the seven transmembrane domain estrogen receptor (G protein-coupled receptor 30) (18,19).

Several human studies have reported significant results regarding the relationship between BPA and genital development. Liu et al (20), in a study that compared maternal, urinary BPA and cord blood sex hormones, maternal urinary BPA was found to be negatively associated with cord blood testosterone levels. The authors proposed that BPA might decrease testosterone levels by affecting both the testes and the pituitary system or by inhibiting the testosterone surge *in utero*. The hypothesis that BPA may reduce testosterone acting as estradiol was also suggested by Nakamura et al (21) in a different study. The effects of endocrine disrupting molecules on androgens are thought to be more profound in the masculinization programming window (8-14 gestational weeks) during intrauterine life, especially in male offspring (22). The findings in our study are coherent with this postulate. 

AGD measurement, as an epidemiological marker of sexual development, is still controversial due to conflicting results from different studies. Miao et al (23) showed a significant relationship between AGD in boys and their parents’ occupational BPA exposure. In a study in adults, Eisenberg et al (24) found a significant association between serum testosterone levels and AGD. However, Parra et al (25) reported no significant relationship between anogenital measurements, reproductive hormone levels and semen quality. 

To our knowledge, this is the first study investigating the relationship between cord blood BPA levels and anogenital measurements in neonatal human subjects. In our study, BPA values in cord blood were found to be higher than those reported in the literature (20,26,27,28). This may be due to poor governmental regulation of water supply companies in our country and the continuous reuse of plastic containers beyond their lifespan. We know that BPA is not the only phenolic endocrine disrupting substance that may be implicated in altering the reproductive development of the fetus, as prenatal exposure to other phenolic molecules and phthalates was also shown to alter AGDs (29). As other environmental pollutants were not measured during our investigation, we can neither confirm nor dismiss their potential adverse effects in our study group. 

AGD is measured in different ways in different studies. AGD_AS_ in boys and AGD_AF_ in girls are mostly accepted as AGDs. We measured the distances from anus to the anterior base of the penis (AGD_AP_) and the base of the scrotum (AGD_AS_) in boys; the distance from anus to the anterior tip of the clitoral hood (AGD_AC_) and the posterior fourchette of labia majora (AGD_AF_) in girls, as described by Sathyanarayana et al (30). The most important finding in our study population was that male newborns in the group with a cord blood BPA level over 90^th^ percentile (8.26 ng/mL) had significantly shorter AGD_AS_ values compared to the group with lower cord blood BPA levels and this finding may reflect the anti-androgenic effect of BPA on fetal male genital development *in utero*. AGD_AC_ was longer in the group with high cord blood BPA levels, although this finding was not statistically significant. The AGD_AF_ did not show any major change with increased levels of BPA. Despite variations in AGDs, none of the patients in our study population had any external genital abnormality which suggests that these changes did not have a major impact on the development of the external genitalia. However, other adverse effects of BPA need to be further investigated.

### Study Limitations

One of the major limitations of our study was the relatively small study sample. Larger series are needed to confirm our results. Another limitation is the high coefficient of variability for which we do not have a clear explanation. 

## Conclusion

The results of our study showed a significant association between high cord blood BPA levels and shortened AGD_AS_ in healthy male newborns. Based on our findings, we suggest that even if BPA has any effect on genital development *in utero*, this effect is subtle at low dose exposure. 

## Figures and Tables

**Table 1:**
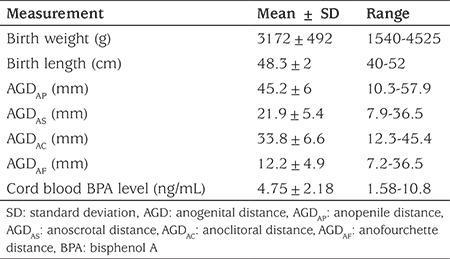
Anthropometric measurements (anopenile distance, anoscrotal distance, anoclitoral distance, anofourchette distance) and cord blood bisphenol A levels

**Table 2:**
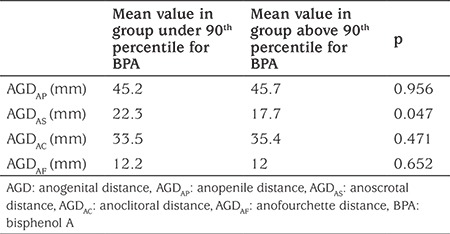
The relationship between anogenital (anogenital distance, anopenile distance, anoscrotal distance, anoclitoral distance, anofourchette distance) measurements and 90^th^ percentile of cord bisphenol A values

## References

[1] Dodds LW (1936). Synthetic estrogenic agents without the phenanthrene nucleus. Nature.

[2] Vandenberg LN, Colborn T, Hayes TB, Heindel JJ, Jacobs DR (2012). , Lee DH, Shioda T, Soto AM, vom Saal FS, Welshons WV, Zoeller RT, Myers JP. Hormones and endocrine-disrupting chemicals: low-dose effects and nonmonotonic dose responses. Endocr Rev.

[3] Rochester JR (2013). Bisphenol A and human health: a review of the literature. Reprod Toxicol.

[4] Brent RL (2013). Bisphenol A and obesity in children and adolescents. JAMA.

[5] Brucker-Davis F, Ferrari P, Boda-Buccino M, Wagner-Mahler K, Pacini P, Gal J, Azuar P, Fenichel P (2011). Cord blood thyroid tests in boys born with and without cryptorchidism: correlations with birth parameters and in utero xenobiotics exposure. Thyroid.

[6] Ferguson KK, Peterson KE, Lee JM, Mercado-Garcia A, Blank-Goldenberg C, Tellez-Rojo MM, Meeker JD (2014). Prenatal and peripubertal phthalates and bisphenol A in relation to sex hormones and puberty in boys. Reprod Toxicol.

[7] Minguez-Alarcon L, Hauser R, Gaskins AJ (2016). Effects of bisphenol A on male and couple reproductive health: a review. Fertil Steril.

[8] Santamaria C, Durando M, Munoz de Toro M, Luque EH, Rodriguez HA (2016). Ovarian dysfunctions in adult female rat offspring born to mothers perinatally exposed to low doses of bisphenol A. J Steroid Biochem Mol Biol.

[9] Seachrist DD, Bonk KW, Ho SM, Prins GS, Soto AM, Keri RA (2016). A review of the carcinogenic potential of bisphenol A. Reprod Toxicol.

[10] Virtanen HE, Adamsson A (2012). Cryptorchidism and endocrine disrupting chemicals. Mol Cell Endocrinol.

[11] Ziv-Gal A, Flaws JA (2016). Evidence for bisphenol A-induced female infertility: a review (2007-2016). Fertil Steril.

[12] Hotchkiss AK, Vandenbergh JG (2005). The anogenital distance index of mice (Mus musculus domesticus): an analysis. Contemp Top Lab Anim Sci.

[13] Hurd PL, Bailey AA, Gongal PA, Yan RH, Greer JJ, Pagliardini S (2008). Intrauterine position effects on anogenital distance and digit ratio in male and female mice. Arch Sex Behav.

[14] Foster PM (2006). Disruption of reproductive development in male rat offspring following in utero exposure to phthalate esters. Int J Androl.

[15] Howdeshell KL, Peterman PH, Judy BM, Taylor JA, Orazio CE, Ruhlen RL, Vom Saal FS, Welshons WV (2003). Bisphenol A is released from used polycarbonate animal cages into water at room temperature. Environ Health Perspect.

[16] Krishnan AV, Stathis P, Permuth SF, Tokes L, Feldman D (1993). Bisphenol-A: an estrogenic substance is released from polycarbonate flasks during autoclaving. Endocrinology.

[17] Tyl RW, Myers CB, Marr MC, Sloan CS, Castillo NP, Veselica MM, Seely JC, Dimond SS, Van Miller JP, Shiotsuka RN, Beyer D, Hentges SG, Waechter JM (2008). Two-generation reproductive toxicity study of dietary bisphenol A in CD-1 (Swiss) mice. Toxicol Sci.

[18] Thomas P, Dong J (2006). Binding and activation of the seven-transmembrane estrogen receptor GPR30 by environmental estrogens: a potential novel mechanism of endocrine disruption. J Steroid Biochem Mol Biol.

[19] Teng C, Goodwin B, Shockley K, Xia M, Huang R, Norris J, Merrick BA, Jetten AM, Austin CP, Tice RR (2013). Bisphenol A affects androgen receptor function via multiple mechanisms. Chem Biol Interact.

[20] Liu C, Xu X, Zhang Y, Li W, Huo X (2016). Associations between maternal phenolic exposure and cord sex hormones in male newborns. Hum Reprod.

[21] Nakamura D, Yanagiba Y, Duan Z, Ito Y, Okamura A, Asaeda N, Tagawa Y, Li C, Taya K, Zhang SY, Naito H, Ramdhan DH, Kamijima M, Nakajima T (2010). Bisphenol A may cause testosterone reduction by adversely affecting both testis and pituitary systems similar to estradiol. Toxicol Lett.

[22] Scott HM, Hutchison GR, Jobling MS, McKinnell C, Drake AJ, Sharpe RM (2008). Relationship between androgen action in the “male programming window,” fetal sertoli cell number, and adult testis size in the rat. Endocrinology.

[23] Miao M, Yuan W, He Y, Zhou Z, Wang J, Gao E, Li G, Li DK (2011). In utero exposure to bisphenol-A and anogenital distance of male offspring. Birth Defects Res A Clin Mol Teratol.

[24] Eisenberg ML, Jensen TK, Walters RC, Skakkebaek NE, Lipshultz LI (2012). The relationship between anogenital distance and reproductive hormone levels in adult men. J Urol.

[25] Parra MD, Mendiola J, Jorgensen N, Swan SH, Torres-Cantero AM (2016). Anogenital distance and reproductive parameters in young men. Andrologia.

[26] Chevalier N, Brucker-Davis F, Lahlou N, Coquillard P, Pugeat M, Pacini P, Panaia-Ferrari P, Wagner-Mahler K, Fenichel P (2015). A negative correlation between insulin-like peptide 3 and bisphenol A in human cord blood suggests an effect of endocrine disruptors on testicular descent during fetal development. Hum Reprod.

[27] Chou WC, Chen JL, Lin CF, Chen YC, Shih FC, Chuang CY (2011). Biomonitoring of bisphenol A concentrations in maternal and umbilical cord blood in regard to birth outcomes and adipokine expression: a birth cohort study in Taiwan. Environ Health.

[28] Fenichel P, Dechaux H, Harthe C, Gal J, Ferrari P, Pacini P, Wagner-Mahler K, Pugeat M, Brucker-Davis F (2012). Unconjugated bisphenol A cord blood levels in boys with descended or undescended testes. Human Reproduction.

[29] Huang PC, Kuo PL, Chou YY, Lin SJ, Lee CC (2009). Association between prenatal exposure to phthalates and the health of newborns. Environ Int.

[30] Sathyanarayana S, Grady R, Redmon JB, Ivicek K, Barrett E, Janssen S, Nguyen R, Swan SH, TIDES Study Team (2015). Anogenital distance and penile width measurements in The Infant Development and the Environment Study (TIDES): methods and predictors. J Pediatr Urol.

